# Wood cauliflower mushroom (*Sparassis crispa)* suppresses the body weight and visceral fat increased by ovariectomy in mice

**DOI:** 10.1016/j.crfs.2024.100713

**Published:** 2024-03-12

**Authors:** Ryoken Aoki, Yasuo Watanabe, Yuki Sakai, Megumi Furukawa, Takahiro Shigetomi, Chen Jiun Rong, Nobuo Izumo

**Affiliations:** a Center for Pharmaceutical Education, Yokohama University of Pharmacy, Yokohama, Japan; b General Health Medical Center, Yokohama University of Pharmacy, Yokohama, Japan; c Laboratory of Pharmacognosy, Yokohama University of Pharmacy, Yokohama, Japan; d School of Nutrition and Health Sciences, Taipei Medical University, Taipei, Taiwan; e Laboratory of Pharmacotherapy, Yokohama University of Pharmacy, Yokohama, Japan; f General Health Medical Research Center, Yokohama University of Pharmacy, Yokohama, Japan

**Keywords:** *Sparassis srispa*, Fruiting body powder, Ovariectomy (OVX), Subcutaneous and visceral fat, Body weight, Lipid metabolism

## Abstract

*Sparassis crispa,* an edible mushroom, has been reported to show many kinds of physiological functions. The present paper focused on reducing body weight, subcutaneous fat, and visceral fat gain in ovariectomized (OVX) mice. Using the fruiting body powder of the indoor cultivation *S. crispa* (IT *S*. *crispa*: ITSc), one week after the OVX, ITSc was administered to two OVX groups by *per os* (p.o). In the sham group, 10 mL/kg water and 10 mL/kg saline were administered by p.o. and *subcutaneous adm*, respectively. OVX groups were divided into four groups. These treatments were performed on animals 6 days a week for 8 weeks. Subcutaneous and visceral fat measurements were performed under inhalation anesthesia with isoflurane using a Latheta LCT-200 X-ray CT system. The biochemical markers and the mRNA expression levels of the PPARγ, adiponectin, TNF-α, PPARα, and leptin were measured. Significant increases in body weight, fat ratio, and glucose levels were detected in OVX mice compared to sham mice. These increases were significantly blocked by ITSc, but not estradiol. Furthermore, ITSc treatment significantly increased adiponectin and leptin levels in adipose tissue. These results suggest that ITSc improves lipid abnormalities due to the less activity of women's ovary function, excluding estrogen functions.

## Graphical abstract

**Figure:**
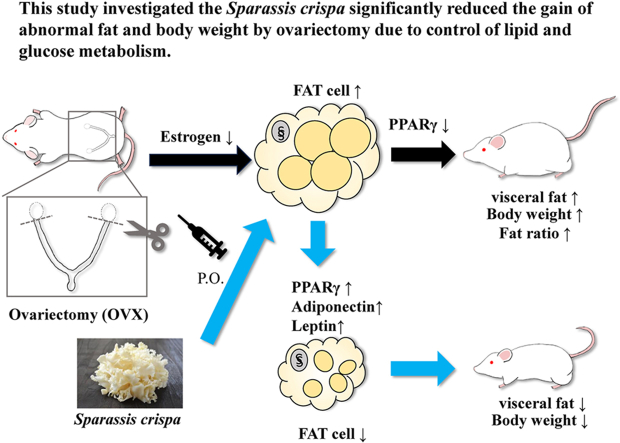

## 1 Introduction

*Sparassis crispa* (kingdom, Fungi; phylum, Basidiomycota; class, Agaricomycetes; order, Polyporales; family, Sparassidaceae; genus, Sparassis; and species, *crispa*) is an edible mushroom and has been used in various dishes for a long time. The safety of this mushroom has been studied in clinical research (Saito et al., 2022). The English name of *S*. *crispa* is Cauliflower mushroom since the appearance of this mushroom is a leaf button-like shape and petal-like aggregates extending from a common base. Since indoor cultivation has become possible and quality is assured, it has been marketed as a nutritional drink and supplement emphasizing multiple functions.

In our recent papers, we summarized the results of a healthy human study using a powdered supplement of the indoor cultivation *S. crispa* (ITSc) reported that ITSc repeated administration reduced several general discomfort symptoms (especially tiredness) during menopause (5 years before and after natural last menstruating) in healthy women (Izumo et al., 2022; Watanabe et al., 2023). The interesting evidence in our clinical results is the lack of direct involvement of endogenous estrogen in the reduction of menopausal symptoms by ITSc. Although previous studies have reported that the mycelia of *S*. *crispa,* which contains brefeldin, shows estrogen-like effects (Kitayama et al., 2018; Dong et al., 2013), brefeldin does not appear present in the fruiting bodies of *S*. *crispa*. On the other hand, it is known that the decrease in estradiol due to menopause also affects fat metabolism, resulting in weight gain due to fat gain (Lizcano and Guzmán., 2014; Kolovou et al., 2014). Regarding the inhibitory effect of *S*. *crispa* on fat gain, Takeyama et al. reported that *S*. *crispa* suppressed body fat mass and triglyceride and cholesterol content in the liver in a study using a high-fat diet-induced male obesity rat model (Takeyama et al., 2018). However, unlike dietary habit-induced obesity, fat gain induced by menopause in women has been reported to be due to abnormal fat metabolism caused by fluctuations in female hormone balance (Tara M D'Eon et al., 2005; Marchand et al., 2018), although *S*. *crispa* has an effect on fat gain at menopause in women has never been studied.

In this study, we investigated the effects and mechanisms of ITSc on body weight, subcutaneous fat, and visceral fat gain in ovariectomized (OVX) mice, an animal model of perimenopausal women.

## 2 Materials & methods

### 2.1 Animals and rearing conditions

Forty ICR female mice (8 weeks old, Japan SLC, Shizuoka, Japan), which had undergone 1 week of preliminary rearing, were used for this study. The breeding environment was set at room temperature (24 ± 1 °C), 12-h light/dark cycles (light period: 07:00–19:00; dark period: 19:00–07:00). Mice were allowed free access to a standard rodent diet (Labo MR stock, Nosan Corporation, Kanagawa, Japan) and water during the entire experimental period. The study was approved by the Animal Experiment Committee of the Yokohama University of Pharmacy (approval number:2022_003), and care was taken for the welfare of laboratory animals.

### 2.2 OVX and administration groups

After 1 week of preliminary rearing, all ICR female mice were divided into 5 groups to minimize the difference in weight. Under inhalational anesthesia with isoflurane, one of the groups (8 mice) underwent sham surgery (sham group), and the other 4 groups underwent ovariectomy (OVX) in 8 animals in each group. One week after the OVX, the fruiting body powder of *S. crispa* (ITSc), donated by Intertrade Healthcare Co., Ltd, Tokyo, Japan (lot number: 200331), was administered. In the sham group, 10 mL/kg water and 10 mL/kg saline were administered by *per os* (p.o.) and *subcutaneous adm* (s.c) respectively. OVX groups were grouped as follows; ①low dosage (10 mg/kg p.o.) of ITSc administration (ITSc10 group) ②high dosage (50 mg/kg p.o.) of ITSc administration (ITSc50 group) ③20 μg/kg s.c. of β-estradiol (Sigma Sigma-Aldrich St. Louis, MO, USA) (E2 group) and ④10 mg/kg s.c. of saline (OVX group). These treatments were performed on animals 6 days a week for 8 weeks.

### 2.3 Measurement of body weight

**Fig. 1:**
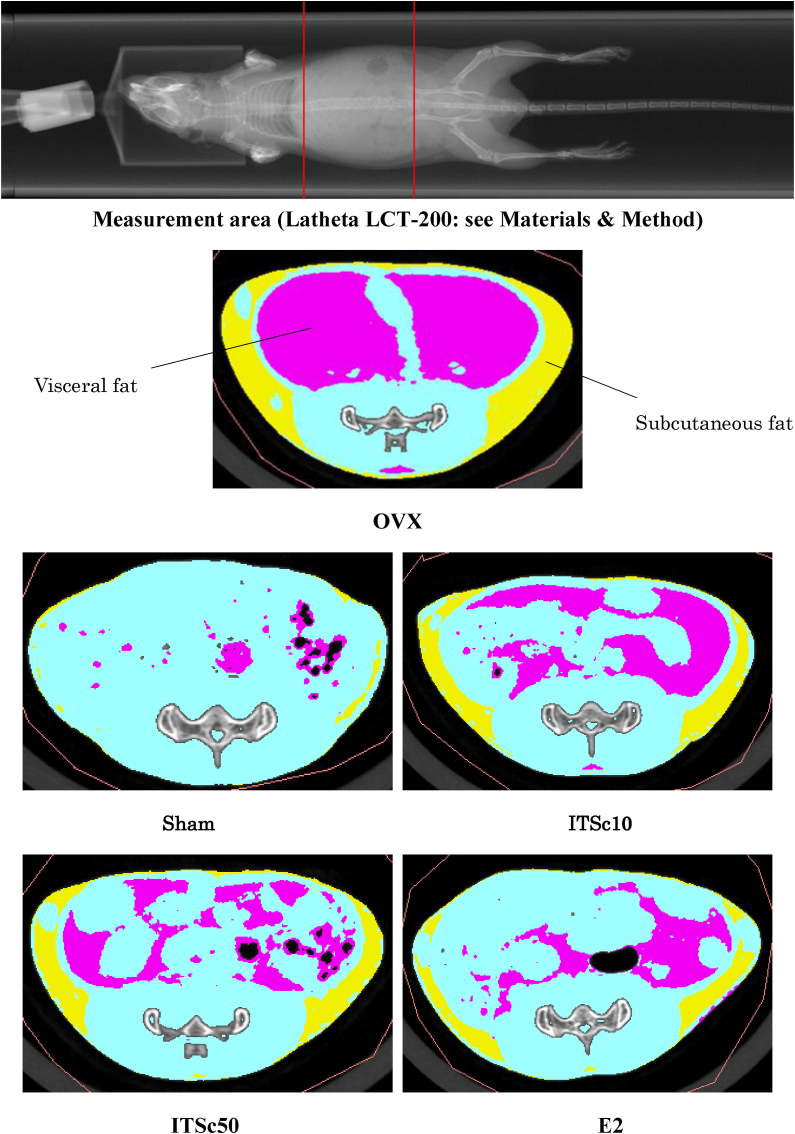
Typical abdomen image in CT imaging.

Body weight changes were measured every experimental day (Aoki et al., 2023). The high-precision electronic balances CJ-2200 (Shinko Denshi Co., Ltd, Tokyo, Japan) measured body weight. In the result, the final body weight at 7 weeks after administration of ITSc (8 weeks after OVX) was shown in Fig. 1.

### 2.4 Measurement of subcutaneous and visceral fats

Subcutaneous and visceral fat measurements were performed under inhalation anesthesia with isoflurane using a Latheta LCT-200 (Hitachi, Ltd., Tokyo, Japan) X-ray CT system for laboratory animals. The imaging conditions were as follows: the voxel size was 96 × 192 μm, the slice thickness was 96 μm, and the slice spacing was 96 μm. The Laboratory CT Latheta ver.3.62 formula calculated each fat volume (gram). The imaging area was from near the diaphragm to the upper pelvis of the mouse.

### 2.5 Biochemical analysis

The blood was collected in heparinized tubes and centrifuged at 3000 rpm at 4 °C for 1.5 min, after which plasma was collected. Plasma concentration of each glucose, triglycerides, cholesterol, and insulin was measured using a Lab Assay ™ Glucose, Triglyceride-E-Test Wako system, Cholesterol-E-Test Wako system (Wako Pure Chemical Corp., Osaka, Japan) and Morinaga mouse Insulin Assay Kits (Morinaga Institute of Bioscience and Technology Corporation, Kanagawa, Japan).

### 2.6 mRNA expression levels of PPARγ, adiponectin, TNF-α, PPARα, leptin

To extract total RNA, Isogen (Nippon Gene, Tokyo, Japan) was added to the Fat specimens extracted from each mouse and homogenized with POLYTRON PT 1300 D (Central Scientific Commerce, Tokyo, Japan). Chloroform (Nacalai Tesque Inc., Kyoto, Japan) was added to the homogenized samples, the samples were centrifuged, and the supernatant was collected in a new tube. Isopropanol (Nacalai Tesque Inc.) was added to the supernatant, and the precipitate was collected by centrifugation. The precipitate was then resuspended in sterile water, the RNA concentration was measured, and cDNA was synthesized using the Super Script VIRO cDNA Synthesis kit (TakaraBio, Tokyo, Japan) protocol, with incubation for 10 min, 37 °C, and 5 s, 85 °C. For real-time PCR, Light Cycler 96 (F. Hoffmann-La Roche, Basel, Switzerland) was used with a primer pair for each marker (Table 1), and the TaqMan probe method was followed for 40 to 50 cycles, where each cycle consisted of 95 °C for 30 s, 95 °C for 5 s, and 60 °C for 30 s.

**Table 1:** List of all primers used for real-time RT-PCR.

GENE	Forward	Reverse
GAPDH	AGTTGTCATCAACGGGAAG	CACCCCATTTGATGTTAGTGG
Adiponectin	ACCGGCAGACAAGAGCAG	TGGTGGGTACAACACCACTC
PPARγ	AGTCCTTCCCGCTGCCAA	GTCGTAGATGACAAATGGTGATTTG
TNF-α	TGCTGGGAAGCCTAAAAGG	CGAATTTTGAGAAGATGATCCTG
PPARα	GCAGTGCCCTGAACATCGA	TGTCGTACACCAGCTTCAGC

### 2.7 Analysis of disaccharides in ITSc

The LC/MS analysis was performed using the JEOL JMS-T100LC AccuTOF system (ESI-negative mode, the range of extracted ion chromatogram (EIC); 341.0000–341.5000). The analytical conditions were as follows: column, Unison UK-Amino (3.0 mm i.d. x 250 mm; 3.0 μm, Imtakt, Kyoto), mobile phase, acetonitrile: 10 mM AcONH4 aq. (3:1); flow rate, 0.3 mL/min; column oven temperature, 40 °C; injection volume, 20 μl. ITSc (250 mg) was extracted with 80% MeOH–H_2_O (40 mL) at 25 °C for 2 h, and then the residue was centrifuged at 3000 rpm for 10 min. The supernatant (10 mL) was diluted with acetonitrile (15 mL) and filtered through a 0.45 μm filter. The resulting solution was used as LC/MS samples.

### 2.8 Statistics

Data are shown as the mean ± standard error. A one-way analysis of variance (ANOVA) was used to identify the differences in all groups. A comparison of the data between groups was performed using Tukey-Kramer Test Stat View (SAS Institute Inc., version 5.0) as statistical software. The significance level was set as p < 0.05 or p < 0.01.

## 3 Results

### 3.1 Effects of ITSc on the Bodyweight

**Fig. 2:**
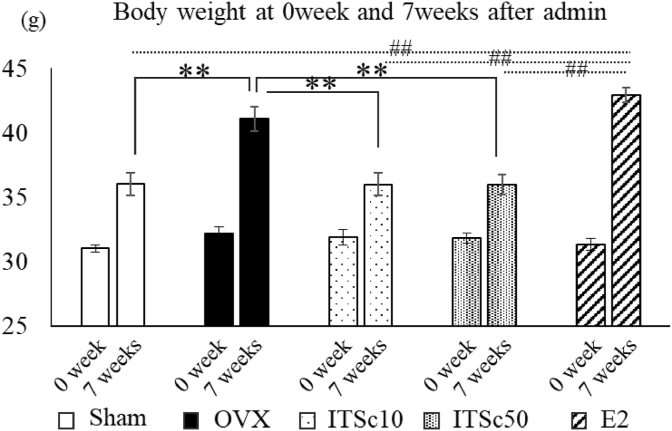
Effects of ITS. crispa（ITSc）and β-estradiol（E2）on body weight of OVX mice. Mean ± S.E. showed the body weight of each group for 0week (pre-administration) and 7weeks after administration. *: p < 0.05 vs OVX, **: p < 0.01 vs OVX, ##: p < 0.01 vs E2, ITSc10：10 mg/kg p.o. of ITS.crispa ITSc50：50 mg/kg p.o. of ITS.crispa E2：20 μg/kg s.c. of β-estradiol.

The body weights at pre-administration (0 week) and 7 weeks after administration of ITSc (8 weeks after OVX) were shown in Fig. 2.

A significant increase in body weight was seen in the OVX group compared to the sham group at 8 weeks after OVX. Both dosages of ITSc administration significantly suppressed body weight gain induced by OVX. Meanwhile, no effect on body weight was observed in the β-estradiol (E2) administration group.

### 3.2 Effects of ITSc on the subcutaneous and visceral fats

**Fig. 3:**
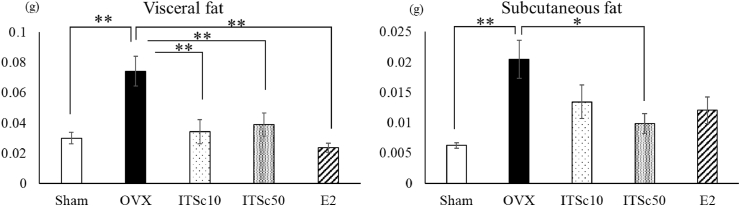
Effects of ITS. crispa（ITSc）and β-estradiol (E2) on Visceral fat and Subcutaneous fat in the abdomen of OVX mice. Mean ± S.E. of each group showed visceral fat and Subcutaneous fat content. *: p < 0.05 vs OVX **: p < 0.01 vs OVX ITSc10: 10 mg/kg p.o. of ITS.crispa ITSc50: 50 mg/kg p.o. of ITS.crispa E2: 20 μg/kg s.c. of β-estradiol.

Eight weeks after OVX, the sham, and OVX groups showed significant increases in visceral and subcutaneous fats in the abdominal region due to OVX (Fig. 3). The regional analysis of fat gain results showed that subcutaneous fat increased 3.3-fold with OVX compared to the sham group (0.0062 ± 0.0004 g). The visceral fat was increased 2.5-fold by OVX compared to the sham group (0.030 ± 0.004 g).

In contrast to the increase in subcutaneous fat induced by OVX (0.020 ± 0.003 g), the ITSc50 group significantly showed a decrease of subcutaneous fat by 51.9 % but not the E2 group (41.1%) (Fig. 3). The ITSc10 group, ITSC50 group, and E2 group significantly decreased visceral fat by 53.9 %, 47.7 %, and 68.1 %, respectively, compared to the OVX group (0.074 ± 0.01 g) (Fig. 3).

### 3.3 Effects of ITSc on the plasma lipid metabolic parameters

**Fig. 4:**
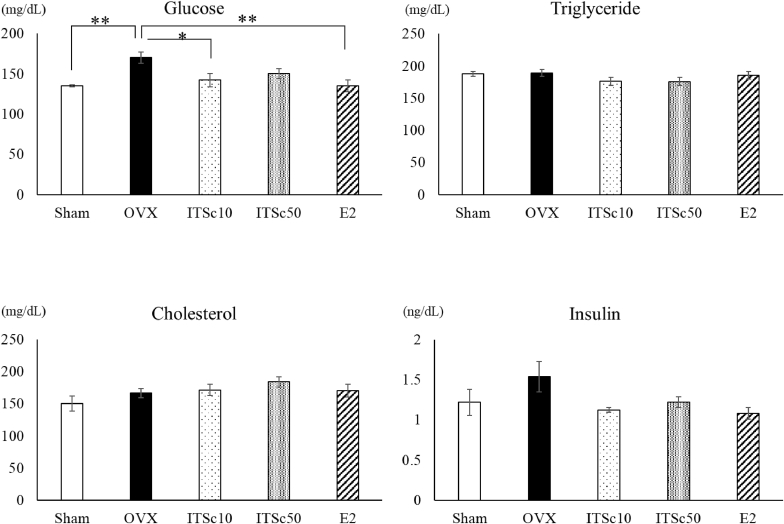
Effects of ITS. crispa（ITSc）and β-estradiol (E2) on Glucose, Triglyceride, Cholesterol, and Insulin in Plasma of OVX mice. Mean ± S.E. of each group showed Glucose, Triglyceride, Cholesterol and Insuline content. *: p < 0.05 vs OVX **: p < 0.01 vs OVX ITSc10: 10 mg/kg p.o. of ITS.crispa ITSc50: 50 mg/kg p.o. of ITS.crispa E2: 20 μg/kg s.c. of β-estradiol.

The effects of ITSc and E2 on glucose, triglyceride, total cholesterol, and insulin levels are shown in Fig. 4. Nine weeks after OVX (8 weeks after administration), blood glucose levels were 135.2 ± 1.4 mg/dL in the sham group, 170.2 ± 7.1 mg/dL in the OVX group, 142.3 ± 8.5 mg/dL in the ITSc10 group, 150.4 ± 5.9 mg/dL in the ITSc50 group, and 135.0 ± 7.2 mg/dL in the E2 group. Glucose levels in the OVX group caused a significant increase (1.3-fold). And this increase in glucose levels was significantly decreased by ITSc and E2 administrations by about 16.5 %, 11.7 %, and 20.8 %, respectively.

Plasma triglyceride levels were 187.9 ± 3.9 mg/dL in the sham group, 189.2 ± 5.1 mg/dL in the OVX group, 176.2 ± 6.3 mg/dL in the ITSc10 group, 176.0 ± 5.9 mg/dL in the ITSc50 group, and 185.7 ± 5.7 mg/dL in the E2 group. The triglyceride levels were decreased by both ITSc administrations by about 7.9 % and 8.0 %, respectively, compared to the OVX group. E2 showed a decreasing trend compared to the OVX group. These data did not show any significant differences (Fig. 4).

Cholesterol and Insulin levels in sham, both ITSc groups and E2 group, did not show any significant changes compared to the OVX group (Fig. 4).

### 3.4 Effects of ITSc on the mRNA expression levels of the PPARγ, adiponectin, TNF-α, PPARα, and leptin

At the end of the test, the mRNA expressions of PPARα, PPARγ, adiponectin, TNF-α, and leptin in adipose tissue were measured using the RT-PCR method. Gene expression was normalized using GAPDH and compared as an expression ratio with the expression of the sham group as 1.

**Fig. 5:**
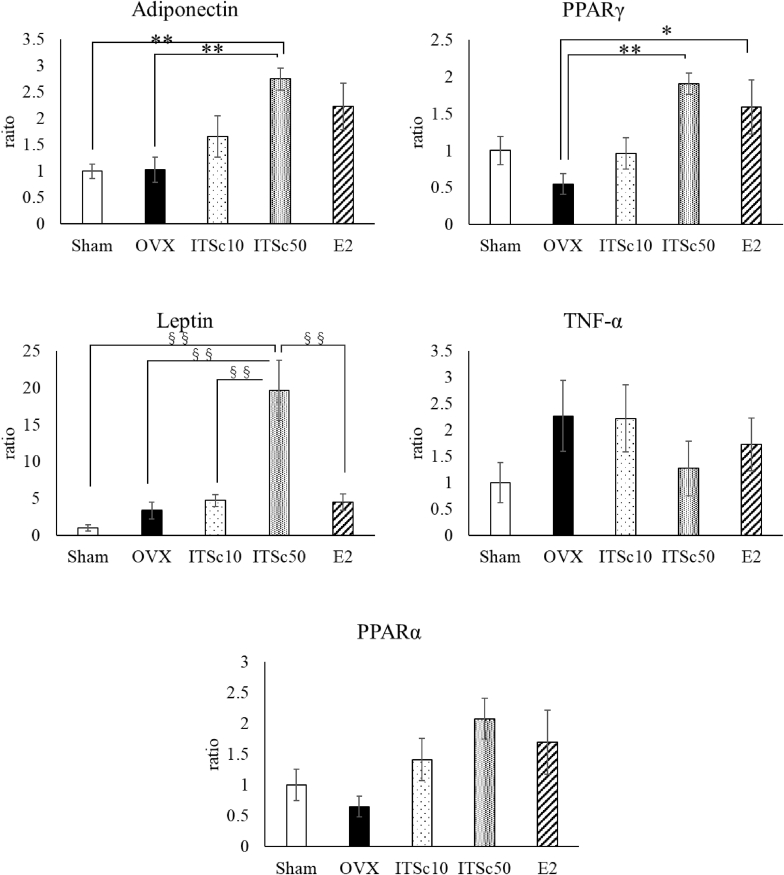
Effects of ITS. crispa（ITSc）and β-estradiol (E2) on PPAR-γ, Adiponectin, TNF-α, PPAR-α and Leptin in adipose tissue of OVX mice. Mean ± S.E. of each group showed PPAR-γ, Adiponectin, TNF-α and Leptin expression. *: p < 0.05 vs OVX, **: p < 0.01 vs OVX, §§: p < 0.01 vs ITSc50 Mean ± S.E. of each group showed PPAR-α expression. **: p < 0.01 vs OVX (Dunnet's analysis but not ANOVA analysis) ITSc10: 10 mg/kg p.o. of ITS.crispa ITSc50: 50 mg/kg p.o. of ITS.crispa E2: 20 μg/kg s.c. of β-estradiol.

The expression ratio of PPARγ was 1.0 ± 0.21 in the sham group, 0.59 ± 0.12 in the OVX group, 0.96 ± 0.21 in the OVX + ITSc10 group, 1.88 ± 0.16 in the OVX + SC50 group and 1.59 ± 0.37 in the E2 group (Fig. 5). The ITSc50 group and the E2 group showed significantly increased expression of PPARγ compared to the OVX group, P < 0.01 and P < 0.05, respectively. These results indicate that ITSc intake increases PPARγ expression, and similar results were obtained with β-estradiol (E2) in the OVX group (Fig. 5).

The expression ratio of adiponectin was 1.0 ± 0.15 in the sham group, 1.04 ± 0.22 in the OVX group, 1.66 ± 0.40 in the ITSc10 group, and 2.66 ± 0.21 in the ITSc50 group respectively (Fig. 5). The ITSc50 group showed significantly increased adiponectin expression compared to the OVX group (P < 0.01). These results indicate that OVX does not alter adiponectin expression, but ITSc50 intake significantly increases adiponectin expression.

The expression ratio of TNF-α was 1.00 ± 0.43 in the sham group, 2.16 ± 0.63 in the OVX group, 2.22 ± 0.64 in the ITSc10 group, and 1.41 ± 0.57 in the ITSc50 group respectively (Fig. 5). The expression of TNF-α in sham, ITSc10, ITSc50, and E2 groups was not significantly different compared to the OVX group.

The expression ratio of PPARα was 1.00 ± 0.29 in the sham group, 0.68 ± 0.17 in the OVX group, 1.41 ± 0.35 in the ITSc10 group, and 1.95 ± 0.35 in the ITSc50 group and 1.70 ± 0.52 in the E2 group (Fig. 5). In the ITSc50 group, PPARα expression was increased compared to the OVX group but not significant. These results indicate that OVX does not alter PPARα expression, but ITSc50 intake has a tendency to increase PPARα expression.

The expression ratio of leptin was 1.00 ± 0.30 in the sham group, 3.19 ± 0.99 in the OVX group, 4.72 ± 0.82 in the ITSc10 group, 21.33 ± 4.23 in the ITSc50 group and 4.48 ± 1.08 in the E2 group (Fig. 5). In the ITSc50 group, leptin expression was significantly increased compared to the OVX group. On the other hand, there was no significant difference in leptin expression between sham and OVX groups. These results indicate that ITSc50 intake increases leptin expression.

### 3.5 Analysis of disaccharides in ITSc by ESI-LC-MS

Disaccharides in ITSc were identified by molecular weight and retention time using LC/MS. In ITSc, trace amounts of disaccharides, except for trehalose, were detected. A few small amounts of disaccharides were sucrose and so on. The trehalose amount was much more than other disaccharides. The trehalose content in ITSc was about 5–10 % (Watanabe et al., 2023).

## 4 Discussion

In this paper, we investigated the effect of ITSc on OVX-induced weight gain and visceral fat gain. There was a statistically significant increase in weight gain in the OVX group compared to the sham group at 8 weeks after ovariectomy (Fig. 2). The increase in body weight after ovariectomy has been reported in previous studies (Jin et al., 2017; Babaei et al., 2010). Our present and previous studies' results confirm other studies' findings (Izumo et al., 2017, 2021; Furukawa et al., 2021). The present study found that continuous forced oral administration of ITSc significantly suppressed OVX-induced weight gain and increase of abdominal fat due to stimulate adipocyte PPARγ system without estrogen pathway.

It has been reported that OVX increases body weight by affecting lipid metabolism through decreased estradiol in vivo (Jin et al., 2017; Babaei et al., 2010). Furthermore, it has been reported that continuous infusion of estradiol suppresses OVX-induced weight gain (Russell et al., 2017). However, in the present study, unlike the results of previous animal studies, no weight loss was observed in the estradiol-treated group. This result may be since estradiol was administered subcutaneously once a day, which may have resulted in a transient increase in estradiol and thus no weight loss effect (Iwasa et al., 2017).

Furthermore, to search for a part of the mechanism of ITSc inhibition of OVX-induced weight gain, we performed a direct visual field search for abdominal fat mass using a CT measuring device for small animals. The results showed a significant increase in fat mass in the OVX group compared to the sham group, and ITSc administration significantly suppressed this increase (Fig. 3). These results suggest that the suppression of OVX-induced weight gain by ITSc may be partly due to the decrease in fat mass. Further detailed examination of subcutaneous and visceral fat was also conducted using a CT scanner. The results showed that the OVX group showed a significant increase in subcutaneous and visceral fat compared to the sham group, and that ITSc treatment had a significant inhibitory effect on the OVX group, especially visceral fat (Fig. 3). Since an abnormal increase in visceral fat is reported to be a risk factor for dyslipidemia and diabetes, it is suggested that ITSc administration may also have a risk-reducing effect on these diseases (Britton et al., 2018; Doyle et al., 2012; Park et al., 2017; Reeves et al., 2011). The present study observed a statistically significant reduction in the subcutaneously administered estrogen and ITSc groups. The estrogen group in this study did not affect weight gain in the OVX-induced group but did have an inhibitory effect on fat mass gain (Fig. 3). Subcutaneous estrogen administration reduces fat mass but does not affect weight loss, suggesting it has pharmacokinetics/pharmacodynamics that is much different from conventional continuous estrogen infusion.

Since fat loss was observed, plasma glucose, triglyceride, and cholesterol concentrations were measured. The plasma glucose concentration was significantly increased in the OVX group compared to the sham group, and the increase in glucose concentration with OVX was reported to be due to decreased sensitivity to insulin caused by the decrease in estradiol (Rettberg et al.,2013). Yamamoto et al. (2010) reported that *S. crispa* lowered blood glucose concentration in a study using KK-Ay mice, a model of type II diabetes, and these results were similar to those obtained with ITSc administration. Since ITSc significantly suppressed the increase in glucose concentration, the plasma insulin concentration was measured. Yamamoto et al. (2010) reported a decrease in insulin concentration in their study. However, our results showed that the ITSc-treated group showed a reduction in insulin levels compared to the OVX group, but the difference was not significant. In the OVX model in this study, there was an increasing trend in insulin concentration compared to the sham group, but the change was not significant. However, the increase in glucose concentration was significant, suggesting that the increase in glucose concentration was not sufficient to induce insulin secretion in this model.

On the other hand, estradiol administration also significantly suppressed the OVX-induced increase in plasma glucose concentration. In those reports, estradiol was also reported to decrease insulin concentrations elevated by OVX (Rettberg et al.,2013).

Plasma triglyceride and cholesterol levels were increased in the OVX group compared to the sham group, but they were not significant. ITSc and estradiol treatment decreased triglyceride levels in the OVX group.

In this study, we measured the gene expression levels of PPARα and PPARγ, which are known to be involved in lipid and glucose metabolism. The results showed that the administration of ITSc significantly increased the gene expression levels of the PPARγ transcription factor compared to the OVX group. These results indicate that administration of ITSc affects lipid and glucose metabolism; Activation of PPARγ has been reported to reduce the size of hypertrophied mast cells (Janani et al., 2014). To investigate the involvement of ITSc in lipid metabolism, we examined the gene expression levels of adiponectin and TNF-α. The results showed a significant increase in adiponectin compared to the OVX group and a decrease in TNF-α. However, there was no significant difference, suggesting that ITSc improves lipid metabolism. Pioglitazone, a drug used in the treatment of diabetes, has been shown to increase adiponectin by acting on PPARγ, which in turn activates AMP kinase, thereby decreasing blood glucose levels (Janani et al., 2014). In the present study, blood glucose levels were also measured, and it was found that ITSc significantly suppressed the increase in blood glucose levels induced by OVX, indicating that ITSc has a PPARγ-mediated hypoglycemic effect.

**Fig. 6:**
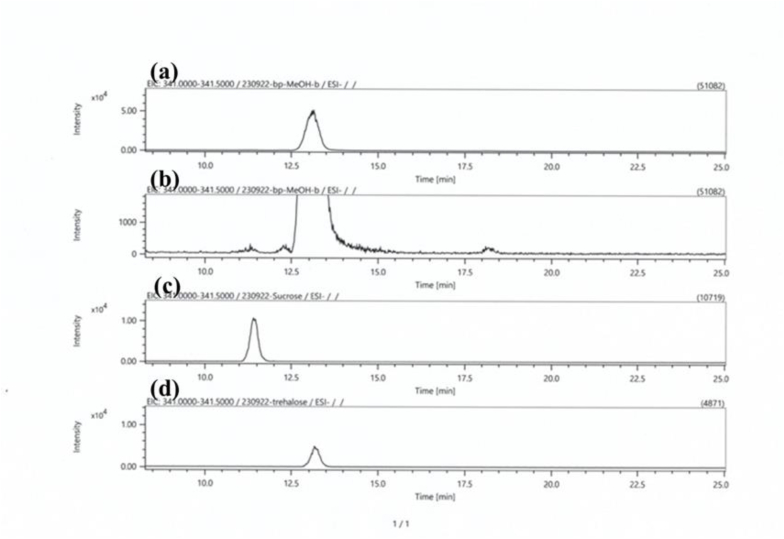
Analysis of disaccharides in ITSc by ESI-LC-MS (A) Extracted ion chromatogram (m/z 341.0000–341.5000) of ITSc extract. (B) Magnified view of intensity of (a) (C) Extracted ion chromatogram (m/z 341.0000–341.5000) of sucrose. (D) Extracted ion chromatogram (m/z 341.0000–341.5000) of trehalose.

The anti-obesity effect of *S. crispa* has been published using high-fat diet rats, and the related ingredients of *S. crispa* have been suggested in more than 30 different materials, including polysaccharides (Takeyama et al., 2018; Sharma et al., 2022). In another previously reported article, trehalose, which is the ingredient of some mushrooms, has an anti-obesity effect (Arai et al., 2013), and ITSc also has disaccharides, particularly trehalose (Fig. 6, Watanabe et al., 2023), suggesting that trehalose is involved in the triglyceride-reducing impact in this study.

The results of the present study suggest that ITSc may act directly on OVX-induced adipocyte gain by upregulating the transcription factors PPARγ and leptin levels, thereby lowering blood glucose levels and inhibiting fat gain. These results suggest that ITSc improves lipid abnormalities due to the less activity of women's ovary function during menopause.

## 5 Conclusions

The consequent oral administration of ITSc, the powder of in-room cultivated *S. crispa,* significantly reduced the gain of abnormal fat and body weight by OVX due to control of lipid and glucose metabolism. These results and our recently published paper suggest that ITSc can be used to control discomforts during or after menopause in women, including the gain of fat and body weight. This paper first showed that *S. crispa* reduces the increase of body weight and abdominal fat induced by OVX using a CT scanner.

## Author Contribution

Aoki R: Conceptualization, Investigation, Writing-original draft preparation. Watanabe Y: Project administration, Conceptualization, Writing, Editing, Funding acquisition. Sakai Y: Project administration, Investigation, Writing. Furukawa M: Project administration, Investigation. Shigetomi T: Investigation, Writing. Chen JR: Project administration, Conceptualization, Editing. Izumo N: Conceptualization, Investigation, Writing-original draft preparation.

All authors have seen and approved the contents of the submitted manuscript.

## Declaration of Competing interest

Although a part of this research has been done using the collaborating research fund of Intertrade Health Co. Ltd., the authors declare no conflict of interest.

## Data Availability

The data that has been used is confidential.
